# Gut and airway microbiota dysbiosis and their role in COVID-19 and long-COVID

**DOI:** 10.3389/fimmu.2023.1080043

**Published:** 2023-03-08

**Authors:** Giuseppe Ancona, Laura Alagna, Claudia Alteri, Emanuele Palomba, Anna Tonizzo, Andrea Pastena, Antonio Muscatello, Andrea Gori, Alessandra Bandera

**Affiliations:** ^1^ Infectious Diseases Unit, Foundation IRCCS Ca’ Granda Ospedale Maggiore Policlinico, Milan, Italy; ^2^ Department of Oncology and Hemato-Oncology, University of Milan, Milan, Italy; ^3^ Multimodal Research Area, Bambino Gesù Children Hospital (IRCCS), Rome, Italy; ^4^ Department of Pathophysiology and Transplantation, Centre for Multidisciplinary Research in Health Science (MACH), University of Milan, Milan, Italy

**Keywords:** microbiota, microbiome, gut-brain-axis, gut-lung-axis, dysbiosis, COVID-19, long Covid, SARS-CoV-2

## Abstract

The gut microbiota plays a crucial role in human health and disease. Gut dysbiosis is known to be associated with increased susceptibility to respiratory diseases and modifications in the immune response and homeostasis of the lungs (the so-called gut-lung axis). Furthermore, recent studies have highlighted the possible role of dysbiosis in neurological disturbances, introducing the notion of the “gut-brain axis.” During the last 2 years, several studies have described the presence of gut dysbiosis during coronavirus disease 2019 (COVID-19) and its relationship with disease severity, SARS-CoV-2 gastrointestinal replication, and immune inflammation. Moreover, the possible persistence of gut dysbiosis after disease resolution may be linked to long-COVID syndrome and particularly to its neurological manifestations. We reviewed recent evidence on the association between dysbiosis and COVID-19, investigating the possible epidemiologic confounding factors like age, location, sex, sample size, the severity of disease, comorbidities, therapy, and vaccination status on gut and airway microbial dysbiosis in selected studies on both COVID-19 and long-COVID. Moreover, we analyzed the confounding factors strictly related to microbiota, specifically diet investigation and previous use of antibiotics/probiotics, and the methodology used to study the microbiota (α- and β-diversity parameters and relative abundance tools). Of note, only a few studies focused on longitudinal analyses, especially for long-term observation in long-COVID. Lastly, there is a lack of knowledge regarding the role of microbiota transplantation and other therapeutic approaches and their possible impact on disease progression and severity. Preliminary data seem to suggest that gut and airway dysbiosis might play a role in COVID-19 and in long-COVID neurological symptoms. Indeed, the development and interpretation of these data could have important implications for future preventive and therapeutic strategies.

## 1 Introduction

### 1.1 COVID-19, long-COVID, and gastrointestinal disease during SARS-CoV-2 infection

Coronavirus disease 2019 (COVID-19) is a highly contagious infectious disease caused by the severe acute respiratory syndrome coronavirus 2 (SARS-CoV-2) virus, a novel RNA beta-coronavirus, with more than 663 million cases and 6.71 million deaths worldwide documented until 20 January 2023 (1). COVID-19 is mainly a respiratory illness, ranging from asymptomatic, mild-moderate, severe, and critical illness (2), especially affecting elderly subjects with underlying medical conditions (3).

After COVID-19, some patients may experience persistent symptoms or other conditions that are colloquially referred to as long-COVID. The *Centers for Disease Control and Prevention* have defined post-COVID conditions as new, returning, or ongoing symptoms that people experience ≥4 weeks after being infected with SARS-CoV-2 (4). The prevalence of these conditions varies widely from 5% to 80%, and the most frequently reported symptoms are fatigue, cough, shortness of breath, and chest pain (2, 5). Furthermore, half of the patients report persistent neurological symptoms at 6 months, the most frequent being “brain fog” and cognitive changes, described in up to one-third of subjects (6).

With regard to the gastrointestinal (GI) tract involvement, early reports from Wuhan showed that 2% to 10% of patients with acute COVID-19 had GI symptoms including nausea and diarrhea (7), but more recent metaanalyses reported a higher prevalence, up to 20% of patients (8). SARS-CoV-2 virus has been detected in anal swabs and stool samples in almost 50% of patients with COVID-19, suggesting that the digestive tract might be an extrapulmonary site for virus replication and activity (9), through ACE2 receptors binding with spike protein-S.

### 1.2 Gut microbiota and its role in health and disease

The human gut microbiota harbors up to 10^14^ resident microorganisms, including bacteria, archaea, viruses, fungi, and other eucaryotes, with bacteria being the most abundant microorganisms at the gut level. The most represented phyla at the gut level are *Firmicutes*, *Bacteroidetes*, *Actinobacteria*, *Proteobacteria*, *Verrucomicrobia*, and *Fusobacteria* (10). An increase in bacteria has been documented from duodenum to colon, with a decrease in facultative anaerobic *Bacilli* (*Firmicutes*) and Enterobacterales (*Proteobacteria*) taxa and an increase in obligate anaerobic bacteria, especially *Bacteroidia* (*Bacteroidetes*) and *Clostridia* (*Firmicutes*) classes (11, 12).

Gut microbiota is crucial for several functions, such as energy extraction from the diet, vitamin and short-chain fat acids (SCFAs) production, and immunomodulation, with the regulation of TH17 and T reg balance (13–15). A complex equilibrium exists among prebiotics, like microbiota accessible carbohydrates (MAC), probiotics, and postbiotics, like their products, SCFAs (16, 17), with involvement of several networks between gut microbiota and other body sites through axes (i.e., gut-lung, gut-liver, gut-brain axis), influencing processes in health and disease.

**Figure 1:**
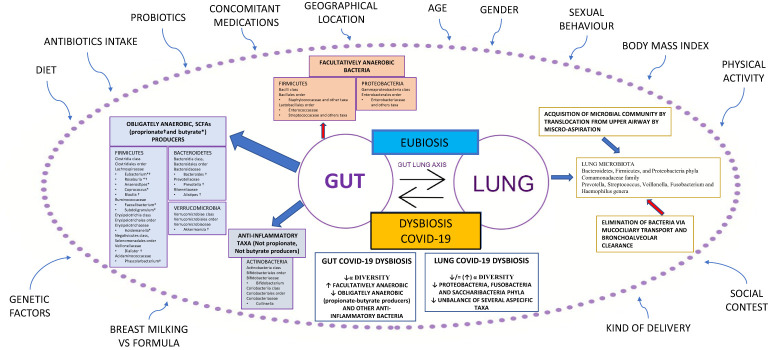
Gut-lung axis microbiota in COVID-19. This figure shows a summary of the gut-lung axis and its alterations during COVID-19. Left: the gut microbiota taxa obligately anaerobic short-chain fatty acids (propionate and butyrate) producers and anti-inflammatory taxa, not propionate and butyrate producers. Upper: facultatively anaerobic bacteria. Right: the homeostasis of the lung microbiota, resulting from acquisition (blue arrow) and elimination (red arrow) clearance. Bottom: the most significant alterations detected in gut and lung microbiota during COVID-19. All around, the confounding factors are strictly related to Microbiota. BMI: Body Mass Index; ° indicates propionate-producing bacteria, * indicates butyrate-producing bacteria, Upward arrows “↑”: increase; Downward arrows “↓”: decrease.

An unbalance of the crucial homeostasis between *Firmicutes*, *Bacteroidetes*, *Actinobacteria*, and *Proteobacteria* phyla (**Figure 1**) is often associated with a change in the numbers of microbes and/or diversity of the microbiota; such a condition is defined as dysbiosis (18). Recently, a new definition of dysbiosis has been suggested, based on a model represented in several diseases, defined by the increase in facultative anaerobic bacteria, like *Bacilli* class and Enterobacterales order, and a parallel decrease in obligate anaerobic bacteria, such as propionate and butyrate-producing bacteria (BPBs) (11).

Gut microbiota dysbiosis can have a role in several disease models affecting the lung, brain, liver, and heart (19).

In the last decade, research on lung microbiota and its pathogenetic link to pulmonary conditions has significantly improved. Previously, the lung has been considered a sterile organ; however, numerous studies have demonstrated the presence of bacterial DNA in the lower respiratory tract in healthy individuals. The lung microbiota of healthy subjects is characterized by the presence of differentiated ecological niches belonging to *Bacteroidetes*, *Firmicutes*, and *Proteobacteria* phyla and *Prevotella*, *Streptococcus*, *Veillonella*, *Fusobacterium*, and *Haemophilus* genera (20). Its balance is the result of acquisition and clearance (**Figure 1**). Many other factors contribute to this complex mechanism, such as the immune system (innate and adaptive immune recognition, secretory IgA), in addition to various exogenous components such as diet, environmental biodiversity, and drug treatments, in particular antibiotics (21).

Chronic respiratory diseases are often characterized by an imbalance between microbial immigration and elimination in the lung. Moreover, the presence of chronic inflammation results in the alteration of physicochemical proprieties that facilitate the growth of select species in the microbial community, such as microorganisms from the *Proteobacteria* phylum, that are linked to a proinflammatory state (22). It is important to emphasize that lung and gut microbiota are in close communication with each other through the circulation of soluble metabolites (i.e., peptidoglycan or LPS) transported by the blood (21). These peptides are recognized by host cells that express pattern-recognition receptors (PRRs), such as Toll-like receptors (TLRs) and Nod-like receptors (NLRs). The interplay between lung and gut microbiota, defined as the gut-lung axis, has been demonstrated in different animal models (23–26).

Further studies are needed to better understand the complex gut-lung interplay and characterize the gut microbial metabolites (i.e., indole derivative, niacin, polyamines, urolithin, and pyruvic acid) that act as immunomodulants and might have a possible impact on respiratory health (27, 28).

Another captivating field of microbiota studies is related to its connection with the brain through the so-called gut-brain axis, which is thought to be a bidirectional system. On one side, there is the involvement of microbiota-derived metabolites on the blood–brain barrier like SCFAs, tryptophan, and linoleic acid metabolites as well as cytokines produced at the gut level; on the other side, the brain controls gut activity through the neuroendocrine and parasympathetic systems (i.e., regulation of intestinal permeability through the vagus nerve) (29). Such connections have been studied in animal models: physiological aging affects gut microbiota in mouse models through cognitive frailty (30).

Gut microbiota dysbiosis seems to play a role in several neurodegenerative and psychiatric disorders (31), as well as in other neurological conditions (32). For example, damage to the GI barrier is a possible pathological pattern for depression disorders; moreover, increased LPS and microbiota-cytokine production seems to be related to Alzheimer’s disease (29).

The relationship between gut microbiota and the brain could be deeper and more complex: alteration of the hypothalamic “master clock” could impact the diurnal environmental fluctuations and lead to dysbiosis-related metabolic disorders like obesity and/or diabetes (33). Furthermore, gut dysbiosis could determine sleep disturbances (sleep loss, alteration of circadian rhythm), eventually leading to fatigue (34). Following this hypothesis, the gut microbiota, which is mostly influenced by diet, could represent a link between the immune and endocrine systems through brain function and the host metabolism (35).

High-fat food intake can indeed damage the GI barrier, affecting both the “intestinal epithelial barrier” (characterized by the mucus layer and the epithelial cells) (36) and the “gut vascular barrier,” regulated by the expression of plasmalemma vesicle-associated protein-1 (PV1). This condition, known as “leaky gut,” can favor microbial translocation to the liver (37), leading to hepatic and systemic disease.

Finally, another example of the role of dysbiosis in disease has been studied in the cardiological setting, where the increased production of trimethylamine (and its metabolite-liver trimethylamine-N-oxide) by gut microbiota has been linked to the development of cardiovascular disease (29).

## 2 Gut microbiota dysbiosis in acute COVID-19

### 2.1 Study characteristics and confounding factors

We identified 22 studies on gut microbiota in COVID-19 patients published in a 2-year window period between 03 January 2020 and 03 January 2022 (**Table 1A**).

**Table 1A:** Selected studies on gut microbiota and COVID-19.

ID	Country	Study characteristics	Population characteristics	α/β-Diversity	Microbiome modifications: relative abundance analyses	Correlations and other findings
Yu et al. (38)	China	Cross-sectional study Anal swab Nanopore-targeted sequencing technology	-2 hospitalized patients are “critical” -Age: 65 and 78 years old -Men: 100% -BMI: no data During ABT and antiviral ongoing lesser comorbidities	No data	**COVID-19 vs. controls** **Relative abundance comparison** ↑ *Actinobacteria* and *Firmicutes* phyla ↑ *Corynebacterium* and *Ruthenibacterium* genera ↓ *Bifidobacterium*, *Lactobacillus*, and *Eubacterium*	The immunologic decline for both patients First report with a link between immune disorders and gut microbiota
Tang et al. (39)	China	Cross-sectional study Stool samples qPCR 10 taxa	-57 hospitalized patients (general, 20 patients; severe, 19 patients; critical disease, 18 patients) -Age (median): 59, 66, 68 -Men: 40%, 47%, 66% -BMI: no data A total of 50.9%, 5.3%, and 12.3% of patients received antibiotics, antifungal drugs, and probiotics, respectively Many patients have more comorbidities especially in critical (hypertension) ones	No data	**Comparison among COVID-19 subgroups** **Relative abundance comparison** ↑ In *Enterococcus* genus/*Enterobacteriaceae* family ratio Ec/E ratio (in critical patients) ↓ *Bifidobacterium*, *Lactobacillus*, *Faecalibacterium prausnitzii*, *Clostridium butyricum*, *Clostridium leptum*, and *Eubaterium rectale*	Correlation between butyrate-producing bacteria (BPBs) and inflammatory markers (PCR, WBC, lymphocyte ratio, neutrophil ratio, IL-6) The first study used the Ec/E ratio to predict death in critically ill patients. The reduction of *Enterobacteriaceae* could be explained using antimicrobial agents active vs. gram-negative.
Zuo et al. (40)	China	Cross-sectional study (15 patients) Prospective study subgroup (5/15) Fecal samples Shotgun metagenomic sequencing	-15 hospitalized patients (2 mild, 8 moderate, 3 severe, 2 critical COVID-19) -Age (median): 55 -Men: 46.6% -BMI: no data 6/15 patient comorbidities (46.7% had stool positivity for SARS-CoV-2. Only 1 patient had diarrhea at presentation)	No data	**Relative abundance comparison** **Patients with high SARS-CoV-2 infectivity:** ↑ *Collinsella aerofaciens*, *Collinsella tanakaei*, *Streptococcus infantis*, *Morganella morganii* **Patients with low-to-none SARS-CoV-2 infectivity:** ↑ *Parabacteroides merdae*, *Bacteroides stercoris*, *Alistipes onderdonkii*, and *Lachnospiraceae bacterium* **Longitudinal arm**: all patients showed substantial variations in fecal microbiome composition regardless of the presence of fecal viral infectivity (confirmed by the longitudinal subgroup)	Patients with low-to-none SARS-COV-2 intestinal replications had a higher abundance of SCFA-producing bacteria Patients with high viral intestinal infectivity have shown an abundance of opportunistic pathogens and higher functional pathways involved in nucleotide metabolism, carbohydrate metabolism, and amino acid biosynthesis
Gu et al. (41)	China	Cross-sectional study Fecal samples V3–V4 of the 16S rRNA gene	-30 hospitalized patients with COVID-19 (15 general, 15 severe COVID-19) 24 hospitalized patients with H1N1 30 matched healthy controls -Age (median): 55 vs. 53 -Men: 56% vs. 56% -BMI: 24.6 vs. 22.9 In the COVID-19 group, 33.3% had at least 1 coexisting medical condition; hypertension (30.0%); 16.7% patients with diarrhea All subjects who received antibiotics, probiotics, or both within 4 weeks before enrollment were excluded.	**α-Diversity** ↓ Shannon diversity Index ↓ Chao-1 diversity Index in COVID-19 and H1N1 patients compared to healthy controls **β-Diversity** No differences between general and severe COVID-19 patients β-Diversity separation according to Bray–Curtis between COVID-19 and controls (and H1N1 subgroup) No β-diversity separation according to the severity index	**LEfSe analysis** **COVID-19 subgroup vs. controls** ↓ *Ruminococcaceae* family, *Fusicatenibacter*, *Anaerostipes*, *Agathobacter*, unclassified *Lachnospiraceae*, and *Eubacterium halli* belong to the *Lacnospiraceae* family) ↑ *Streptococcus* genus No differences between general and severe COVID-19 patients	COVID-19-related genera: *Streptococcus*, *Rothia*, *Veillonella*, *Erysipelatoclostridium*, and *Actinomyces* Control group-related genera: *Romboustia*, *Faecalibacterium*, *Fusicatenibacter*, and *Eubacterium halli* group *Agathobacter*, *Fusicatenibacter*, *Roseburia*, and *Ruminococcaceae* −Correlated with CRP, PCT, or D-dimer levels CRP and D-dimer levels + correlated with COVID-19-enriched bacteria Significant depletion of BPB in the COVID-19 cohort
Tao et al. (42)	China	Cross-sectional Fecal samples V4 of the 16S rRNA gene	-26 patients COVID-19 33 influenza patients 40 controls Clinical information not shown	**α-Diversity** ↓Chao-1 **β-Diversity** The Unifrac-weighted separation between COVID-19 and controls	**LEfSe analysis** **COVID-19 vs. controls** ↑ *Streptococcus*, *Clostridium*, *Lactobacillus*, and *Bifidobacterium* genera ↓ *Bacteroides*, *Roseburia*, *Faecalibacterium Coprococcus*, and *Parabacteroides*	+Correlation IL-18 and gut marker and *Peptostreptococcus*, *Fusobacterium*, and *Citrobacter* taxa −Correlation between *Bilophila* and *Citrobacter* genera and disease severity *Streptococcus* genus
Zuo et al. (9)	China	A prospective study (short-term, from admission until discharge) Fecal samples Shotgun metagenomic sequencing	-15 hospitalized patients with COVID-19 (1 mild, 9 moderate, 3 severe, 2 critical) 6 hospitalized patients with pneumonia 15 healthy individuals -Age: 55 median (COVID-19), 48-year-old median controls -Men: 47% (COVID-19) vs. 60% (controls) -BMI: no data 40% of patients with COVID-19 had comorbidities, especially hypertension, hyperlipidemia, and diabetes mellitus (only 1 patient had diarrhea) Antibiotic used: amox/clav, cephalosporin, tetracyclin 7/15 patients ABT naïve Antiviral therapy: LPV/RTV; ribavirin, INFbeta-1b	**α-Diversity** No data **β-Diversity** Bray–Curtis dissimilarity between antibiotic-naive patients, patients who received antibiotics, and controls	**Relative abundance comparison** **Antibiotic-naïve subgroup vs. controls:** ↑ *Actinomyces viscosus*, *Clostridium hathewayi*, and *Bacteroides nordii* ↓ *Eubacterium ventriosum* **In the antibiotic subgroup:** ↓ *Eubacteriaceae* and *Ruminococcaceae families* ↓ *Blautia, Eubacterium*, *Faecalibacterium*, *Roseburia*, and *Coprococcus* genera ↓ *Dorea formicigenerans*, *Faecalibacterium prausnitzii*, *Eubacterium rectale*, *Ruminococcus obeum*, *Lachnospiraceae bacterium*, and *Eubacterium ventriosum* species 23 bacterial taxa were found to be significantly associated with COVID-19 disease severity: ↑ *Erysipelotrichia* class ↑ *Erysipelotrichales* order ↑ *Erysipelotrichaceae* family ↑ *Coprobacillus*, *Enterobacter* genera ↑ *Clostridium ramosum*, *Clostridium hathewai*, *Erysipelotrichaceae noname*, *Actinomyces odontolyticus*, *Erysipelotrichaceae bacterium*, *Enterobacter cloaceae*, *Parabacteroides* unclassified, and *Alistipes indistinctus* species ↓ *Dorea*, *Roseburia*, and *Faecalibacteirum* genera ↓ *Bifidobacterium pseudocatenulatum*, *Dorea longicatena*, *Bacteroides ovatus*, *Anaerostipes hadrus*, *Lachnospiraceae* bacterium, *Faecalibacterium prausnitzii*, and *Alistipes onderdonkii*	*Clostridium ramosum* and *Clostridium hathewayi* were +associated with COVID-19 disease severity. *Alistipes onderdonkii* and *Faecalibacterium prausnitzii* showed a −correlation with COVID-19 severity 14 Bacterial species associated with a fecal viral load of SARS-CoV-2: -*Bacteroides dorei*, *Bacteroides theteiotaomicron*, *Bacteroides massilinesis*, and *Bacteroides ovatus* showed significant −correlation with fecal SARS-CoV-2 load *Erysipelotrichaceae* bacterium showed the strongest +correlation with fecal SARS-CoV-2 load Antibiotic treatment in patients with a more heterogeneous microbiome configuration In antibiotic-naive patients with COVID-19 ↑ opportunistic pathogens ↓ multiple bacterial species, which are symbionts beneficial COVID-19 condition the strongest factor on gut microbiota followed by hyperlipidemia, pneumoniae, and antibiotics Gut dysbiosis persistence over time regardless of clearance of SARS-COV-2 The link between gut dysbiosis and the expression of ACE2: possible role of *Firmicutes* members to upregulate ACE2-R expression; possible role of *Bacteroidetes* members to downregulate ACE2-R expression
Yeoh et al. (43)	China	2 Hospital cross-sectional study Longitudinal arm subgroup 30 days after virological clearance Fecal samples Shotgun metagenomic sequencing	100 Hospitalized patients with COVID-19 (mild, 45; moderate, 45; severe, 5; critical, 3) 78 controls -Age: 36 vs. 45 years old -Men: 53% vs 42% -BMI: no data ABT: 34 patients Antivirals: 46 patients prior to stool collection (LPV/RTV, ribavirin, oseltamivir) Comorbidities: hypertension, hyperlipidemia, diabetes, and heart conditions (17% diarrhea at admission) For control hypertension	**α-Diversity** No significant differences in species richness and Shannon diversity between COVID-19 and controls **β-Diversity** Separation among COVID-19 with antibiotics, without antibiotics, and controls After virological cure, gut microbiota remained significantly distinct at 30 days (more dissimilar composition in patients who had received antibiotics)	**Relative abundance comparison** **COVID-19 vs. controls** ↑ *Bacteroidetes* phylum *Ruminococcus gnavus*, *Ruminococcus torques*, *Bacteroides dorei* species ↓ *Actinobacteria* phylum ↓ *Bifidobacterium adolescentis*, *Faecalibacterium prausnitzii*, and *Eubacterium rectale* species After antibiotic effects evaluation: ↑ *Parabcteroides* genus ↑ *Sutterella wadsworthensis*, *Bacteroides caccae* species ↓ *Adlercreutzia equolifaciens*, *Dorea formicigenerans*, *Clostridium leptum* species	−Correlation in *Faecalibacterium prausnitzii* and *Bifidobacterium bifidum* with severity +Correlation in CXCL10, IL-10, TNF-α, AST, GGT CRP, LDH, NT-proBNP, and erythrocyte sedimentation rate with microbiota composition Microbiota distribution was associated with COVID-19 and antibiotics but not with stool SARS-CoV-2 viral replication, antiviral, corticosteroids, and pomp inhibitor use. Continuum PCA visualization of a gut microbial composition according to severity index disease Postulated that gut microbiota was associated with the magnitude of immune response to COVID-19
Mazzarelli et al. (44)	Italy	Cross-sectional monocenter study Anal swab V2, V4, V8, and V3–6, 7–9 of the 16S gene	-15 hospitalized inpatients (9 in the ward w-COVID-19, 6 intensive cure unit, i-COVID-19) 8 hospitalized inpatient controls (3 in the intensive care unit, 5 on the floor) Severity: not possible stratification; all patients (including controls) pneumonia -Age: 67 (ward), 70 (ICU), 69 controls -Men: 55%, 50%, and 62%, respectively, in wards, ICU, and controls -BMI: no data ABT: 55%, 50%, and 37%, respectively, in the ward, ICU, and controls 48% antibiotics 1 or 2 days before the anal swab	**α-Diversity** ↓ Chao-1 Trend ↓ Shannon diversity index **β-Diversity** According to Bray–Curtis distinct patterns among the 3 groups	**Relative abundance comparison** **w-COVID-19 vs. controls** ↑ *Proteobacteria* phylum ↑ *Peptostreptococcaceae*, *Enterobacteriaceae*, *Staphylococcaceae*, *Vibrionaceae*, *Aerococcaceae*, *Dermabacteraceae* families, *Actinobacteria* taxa ↓ *Spirochaetes* and *Fusobacteria* phyla ↓ *Nitrospiraceae*, *Propionibacteriaceae*, *Aeromonadaceae*, *Moraxellaceae*, and *Mycoplasmataceae* families **w-COVID-19 vs. i-COVID-19:** ↑ *Carnobacteriaceae*, *Peptobacteriaceae*, *Moritellaceae*, *Selenomonadaceae*, *Micromonosporaceae*, and *Coriobacteriaceae* families ↓ *Staphylococcaceae*, *Microbacteriaceae*, *Micrococcaceae*, *Pseudonocardiaceae* families; *Erysipelotrichales* taxa **i-COVID-19 vs. CTRL:** ↑ *Staphylococcaceae*, *Aerococcaceae*, *Dermabacteraceae*, *Erysipelotrichaceae*, *Microbacteriaceae*, *Mycobacteriaceae, Pseudonocardiaceae*, *Brevibacteriaceae* families; *Actinobacteria* taxa ↓ *Carnobacteriaceae*, *Coriobacteriacea*e, and *Mycoplasmataceae* families	High levels of ferritin detected in i-COVID-19 patients in comparison to w-COVID-19 ↓ Of SCFA-producing bacteria A distinct profile can be distinguished between i-COVID-19 and w-COVID-19 with the latter being closer to CTRL.
Liu et al. (45)	China	Prospective, interventional, single-centered pilot study on fecal microbial transplantation (FMT) Fecal samples before and after 1 week of FMT 16S sequencing	11 COVID-19 patients 1-month after a hospital discharge form -Age: 50 average -Men: 6/11 (54%) -BMI: no data 10 patients non-severe, 1 patient severe No antibiotics or an anti-inflammatory drug for 2 weeks prior to the treatment 5 out of 11 patients suffered from GI	**α-Diversity** -6 months ↑ Chao-1 after TMT No differences with other indexes (Shannon, Simpson, observed, OUT num) **β-Diversity** No data	**Relative abundance comparison** **Before vs after 1 week of FMT** ↓ *Proteobacteria* ↑ *Actinobacteria* ↑ *Bifidobacterium*, *Faecalibacterium*, and *Collinsella* genera	FMT effect on B lymphocytes ↓ naïve B cells, ↑ memory B cells, and non-switched B cells Alleviated GI symptoms were observed after FMT. First intervention study with FMT in a COVID-19 setting
Xu et al. (46)	China	Prospective study 35 days after symptomatic resolution Throat samples and anal swabs V4 region of bacterial 16 S rRNA gene	-35 COVID-19 patients, 19 healthy controls 10 non-COVID-19 patients with other diseases 34/35 COVID-19 patients with mild symptoms -Age: 47 average -Men: 57% -BMI: no data ABT: 13/35 37%, essentially fluoroquinolones 1 patient receiving steroids 14 patients receiving oseltamivir or INF Comorbidities: 16/35, with hypertension, more representative	**α-Diversity** ↓ Richness (observed) and Evenness (Pielou’s evenness) indexes from types I to III during the early phase of COVID-19 **β-Diversity**: according to Bray–Curtis, 3 microbial community types were identified (types I–III)	**Dirichlet multinomial mixture (DMM) clustering: comparison among groups** Type I: *Bacteroide*s genus and several known butyrate-producing bacteria: *Faecalibacterium*, *Roseburia*, *Blautia*, and *Coprococcus* genera; 1 opportunistic pathogenic bacterium *Finegoldia* genus Type II: *Neisseria*, *Actinomyces*, and others Type III: *Pseudomonas* genus members -A shift of the gut microbiome from the lower-diversity community type (II or III) toward a higher-diversity type (I or II) was observed over time in 7/10 patients who had anal swab tests at different timepoints -Clear trend of increased bacterial diversity and the relative abundance of *Bacteroides* and *Faecalibacterium* from early to late stages of COVID-19 like restoration of gut microbiota	Respiratory microbiome: α-Diversity decreased from type I to type IV. Except for the duration of COVID-19, the upper respiratory and gut microbial community divergence seemed not to be associated with age, gender, antibiotics use, and detection of SARS-CoV-2 RNA (the use of antibiotics could emphasize both dysbioses) The shift of microbiome community types over time appeared to match between the throat and the gut in 6/8 patients −Correlation α-diversity with serum LPS Dysbiosis of the upper airways seems to appear early and worse compared to the gut, due to a different resilience status in association with a high permeability among organs due to inflammation.
Ren et al. (47)	China	Cross-sectional study -Fecal samples and tongue-coating samples V3–V5 region of the 16S rRNA gene	The discovery cohort: CPs: 24 fecal samples 48 tongue-coating samples HCs: 48 fecal samples 100 tongue-coating samples -Age: 48 years old, 48 years old for controls -Men: 28% vs. 8% -BMI: not calculated Severity index not calculated: probably mild No clinical information about comorbidities	**α-Diversity** ↓ Observed richness and evenness/diversity index (Shannon index) **β-Diversity** PCoA separation among groups	**Relative abundance comparison** **Comparison between COVID-19 and controls** ↓ *Pseudobutyrivibrio*, *Ruminococcaceae* uncultured, *Blautia*, *Faecalobacterium*, *Bacteroides*, *Akkermansia*, *Lachnospiraceae incertae sedis*, and *Bifidobacterium* taxa ↑ *Streptococcus* and *Enterococcus* genera The article described 5 reduced genera (*Faecalibacterium*, *Lachnospira* genera, and others) and 5 increased genera (not specified)	Oral microbiome alterations: α-Diversity → Shannon index and Simpson index significantly decreased in the CPs vs. HCs β-Diversity → Significant distinction of oral microbial communities between both groups
Chen et al. (48)	China	Prospective study: 6 months follow-up Fecal samples V3–V4 of the 16S rRNA gene	-30 patients subdivided post-convalescence phase using the median Chao-1 cutoff 259 in low α-diversity (*N* = 15), high α-diversity (*N* = 15) -Acute phase (from illness onset to viral clearance) -Convalescence (from viral clearance to 2 weeks after hospital discharge) -Post-convalescence (6 months after hospital discharge) 30 control patients -Age: 53 -Men: 63% -BMI: 24 33.3% severe illness	**α-Diversity** ↓ Richness Chao-1 Index in the acute phase compared to controls Richness was not restored to normal levels after 6-month recovery (trend toward controls) **β-Diversity** A Bray–Curtis analysis separation between COVID-19 and controls	Abundance relative analysis was not performed	Patients with reduced post-convalescence richness had higher levels of CRP as well as a higher occurrence of ICU admission and HFNC during the acute phase. In post-convalescence, low richness was associated with reduced FVC, FEV1, inspiratory vital capacity, and total lung capacity. Post-convalescence patients with lower microbial richness had worse pulmonary functions. Patients with lower richness at 6 months had an illness severity during the acute phase with a strong link between inflammatory response and COVID-19 gut microbiota dysbiosis.
Gaibani et al. (49)	Italy	Cross-sectional multicentered study Fecal samples V3–V4 of the 16S rRNA gene	-69 COVID-19 control patients: healthy age-gender-therapy and hospitalization-related confounder-matched (like exposure to antibiotics 2 weeks before: 69%) Italians For a subanalysis, a non-COVID-19 in ICU controls matched for age, gender, antibiotics, and other factors -Age: 73 -Men: 55% -BMI: 24 median (16% with obesity); 22–27 IQR 77% presented with moderate/severe pneumonia during hospitalization: 33% severe respiratory failure, 23% ICU, and 14% mechanically ventilated Hydroxychloroquine, low-molecular-weight heparin (LMWH): 88.4% Tocilizumab: 36% DRV; DRV/Cobi: 4.4%, 7.2% Several comorbidities: hypertension, 63%; COPD, 22%; diabetes, 17%; and others	**α-Diversity** ↓ Evenness index (inv.Simpson index) **β-Diversity** According to Bray–Curtis, the a significant separation between COVID-19 patients and healthy controls. Note: gut microbiota profiles of COVID-19 patients showed no segregation by age, sex, antibiotic intake in the 2 weeks prior to fecal sampling, length of hospital stay, the time interval between fecal sampling, length of hospital stay, the time interval between fecal sampling and hospital admission, and outcome (death/discharge).	**LefSe analysis** **COVID-19 patients vs. controls** ↑ *Enterococcaceae*, *Coriobacteriaceae*, *Lactobacillaceae*, *Veillonellaceae*, *Porphyromonadaceae*, *Staphylococcaceae*, and *Eysipelotrichaceae* families ↑ *Enterococcus*, *Lactobacillus*, *Collinsella*, *Staphylococcus*, *Akkermansia*, *Parabacteroides*, *Actinomyces*, *Serratia*, *Lactococcus*, *Phascolarbacterium*, *Odoribacter*, *Acidaminococcus*, and *Methanobrevibacter* genera ↓ *Bacteroidaceae*, *Lachnospiraceae*, *Ruminococcaceae*, *Prevotellaceae*, and *Clostridaceae* families ↓ *Prevotella*, *Bacteroides*, *Faecalibacterium*, *Coprococcus*, *Blautia*, *Ruminococcus*, *Erwinia*, *Oxalobacter Roseburia*, *Anaerofustis*, *Lachnospira*, *Scardovia*, *Anaerofilum*, *Dialister*, *Oscillospira*, *Holdemania*, *Cloacibacillus*, and *Cristensenella* genera Note: sequences assigned to *Enterococcus* were *E. faecium* (8.4%) along with *E. hirae* (5.5%), *E. faecalis* (1.8%), and *E. villorum* (1.1%) ↑ *Enterococcus* in ICU patients and those developing BSI. ↑ *Streptococcus*, *Oscillospira*, *Blautia*, and other *Ruminococcaceae*, *Lachnospiraceae*, and *Clostridiales* taxa in patients who had not entered the ICU and those who had not developed BSI	The severity of COVID-19-related dysbiosis is strongly associated with the development of BSI and ICU admission The percentage of patients who developed E-BSI was significantly higher during the COVID-19 pandemic than in the previous 3 years. Due to the severity of the clinical setting of the population, they could not exclude previous antibiotic intake before ICU admission, but controls were matched also for this parameter After an intragroup comparison between patients ICU admitted vs. patients COVID-19 no-ICU admitted, they did not see α-diversity differences but only a β-diversity separation among groups (including ICU controls). Both COVID-19 subgroups (ICU and no-ICU) expressed high levels of *Enterococcus* species compared to ICU controls No-ICU COVID-19 had an overrepresentation of *Ruminococcus*, *Oscillospira*, *Dorea*, and *Coprococcus*. ICU controls had an overrepresentation of *Enterobacteriaceae* (in particular, *Klebsiella* species)
Zhou et al. (50)	China	Cross-sectional study -Fecal samples -Shotgun metagenomic sequencing	-187 COVID-19 patients (127 patients with fever and 60 patients with no fever). All moderate COVID-19 -Age: 39 median (37 in the fever subgroup vs. 48 in the no-fever subgroup) -Men: 34% (36% vs. 31%) -ABT: No data Several comorbidities especially hypertension	**α-Diversity** ↓ In patients with fever with a strong trend according to Chao-1 (not significant according to Shannon) **β-Diversity** According to Bray–Curtis, different compositions in the gut microbiota between the 2 groups	**LEfSe analysis** **Fever group vs. no-fever group** ↑ *Ascomycota* phylum (fungal) ↑ *Saccharomyces* (fungal) and *Enterococcus* genera ↑ *Enterococcus faecalis*, *Citrobacter freundii*, *Citrobacter* unclassified, *Haemophilus parainfluenzae*, and *Saccharomyces cerevisiae* species ↓ *Bacteroidetes* phylum ↓ *Anaerostipes*, *Prevotella, Parabacteroides*, *Phascolarbacterium*, *Eggerthella* genera ↓ *Bacteroides cellulolyticus*, *Bacteroides fragilis*, *Bacteroides thetaiotaomicron*, *Bacteroides xylanisolvens*, *Eubacterium ramulus*, and *Erysipelotrichacae bacterium*	Patients with fever: more pathogens, and lack butyrate-producing species. 5 epitopes were enriched in the fever group. Some of these were +correlated with clinical indices (IL-6, WBC, neutrophils, CRP, D-dimer, and LDH). 4 of the 5 epitopes were all +correlated with *E. faecalis* (↑ in the fever group). Same background, although during ABT treatment and with no available diet investigation information
Kim et al. (51)	South Korea	Prospective monocenter study 2 time points: from positive to negative virological cure Fecal samples V3–V4 of the 16S rRNA gene	12 out-patients Longitudinal analysis from positive (infected state) to negative virological test (recovered state) 36 controls Asymptomatic infection or mild COVID-19 -Age: 26 -Men: 66% -BMI: 23 No medicines and/or antibiotics and/or probiotics ongoing Few comorbidities but gastrointestinal tract involvement (reflux esophagitis, irritable bowel disease, fatty liver)	**α-Diversity** ↑ Evenness index in the recovered state (Pielou’s evenness) (the trend for Shannon; not for richness indexes like faith and observed) trend toward controls **β-Diversity** Differences for quantitative indexes Bray–Curtis and weighted Unifrac (respectively phylogenetic and no-phylogenetic measures). No differences for qualitative indexes Jaccard and unweighted Unifrac (respectively no-phylogenetic and phylogenetic measures) trend toward controls	**Relative abundance comparison** **Infected state vs. recovered state** ↓ *Bacteroidetes, Bacteroidia*, *Bacteroidales*; *Bacteroidaceae*, *Marinifilaceae*, and *Tannerellaceae* families ↑ *Actinomycetals* order, *Actinomyces* order **COVID-19 vs. controls** ↓ SCFA-producing bacteria and *Bacteroides*, *Butyricimonas*, and *Odoribacter* taxa and members of *Lachnospiraceae* and *Ruminococcaeae* families	↑ *Firmicutes*/*Bacteroidetes* ratio in an infected state, in the absence of antimicrobial therapy and without obese patients +Correlation between *Escherichia*/*Shigella*, *Citrobacte*r, *Collinsella*, and *Bifidobacterium* and COVID-19
Zhou et al. (52)	China	Cross-sectional prospective study of recovered COVID-19 healthcare workers (HCWs) after 3 months Fecal samples V3–V4 of the 16S rRNA gene	-15 HCWs, 14 controls 80% had at least 1 long COVID-19-related symptom (especially cough and fatigue) -Age: 29 medians vs. 37 controls -Men: 20% vs. 35% in controls -BMI: 22 vs. 24 2 recovered HCWs with hypertension; no comorbidities in the controls Excluded patients with previous antibiotics and/or probiotics within 3 months before enrolment (no information on lifestyle/diet)	**α-Diversity** **HCWs vs. controls** ↓ with Shannon (and not sign with other indexes) **β-Diversity** According to Bray–Curtis, a significant difference in the fecal microbiota between recovered HCWs and HCs	**Relative abundance comparison** **HCWs vs. controls** ↑ *Actinobacteria* phylum ↑ *Escherichia*, *Flavonifractor*, and *Intestinibacte*r genera ↑ *Esherichia* unclassified, *Intestinibacter bartlett*, *Clostridium aldenense*, *Clostridium bolteae*, *Flavonifractor plautii*, and *Clostridium ramosum* species ↓ *Lachnospiraceae*, *Desulfovibrionaceae* families ↓ *Faecalibacterium*, *Roseburia*, *Fusicatenibacter*, *Ruminococcus*, *Clostridium XVIII*, *Dorea*, *Butyricicoccus*, *Romboutsia*, *Intestinimonas* and *Bilophila* genera ↓ *Faecalibacterium prausnitzii*, *Roseburia inulinivorans*, *Fusicatenibacter saccharivorans*, *Ruminococcus bromii*, *Blautia faecis*, *Butyricicoccus pullicaecorum*, and *Intestinimonas butyriciproducens* species	−Correlation between *Faecalibacterium prausnitzii* and chest tightness after activity −Correlation between *Intestinimonas butyriproducens* and cough +Correlation between *Escherichia* unclassified and fatigue, chest tightness after activity, and myalgia +Correlation between *Intestinobacter bartlettii* and anorexia and fatigue Compared with HCs, the fecal microbiota of recovered HCWs at 3 months after discharge exhibited decreased bacterial diversity
Moreira-Rosario et al. (53)	Portugal	Multicenter cross-sectional study Fecal samples V3–V4 of the 16S rRNA gene	-115 COVID-19 patients Severity index: 19 mild, 37 moderate, 59 severe Location: 14 ambulatory, 40 wards, 61 ICU -Age: 68 median -Men: 63% -BMI: not shown, percentage of overweight or obese: 65% Comorbidities: hypertension, diabetes, and other ABT: 38% during the last 6 months	**α-Diversity** Decrease trend for α-diversity Shannon index (diversity index) from mild to severe. **β-Diversity** No data	**Relative abundance comparison** **Mild COVID-19 vs. moderate COVID-19 and mild COVID-19 vs. sever COVID-19:** Decrease tendency from mild to moderate and from moderate to severe for: *Bifidobacteriaceae* (*Bifidobacterium* genus) and *Coriobacteriaceae* (*Collinsella* genus) taxa with significant differences ↓ for *Lachnospiracea*e family (*Roseburia* and *Lachnospira* genera) ↑ *Ralstonia* genus (*Proteobacteria* phylum) with COVID-19 severity score index *Firmicutes*/*Bacteroidetes* ratio has decreased through severity increase	In a multivariate analysis, the Shannon index and CRP were associated with COVID-19 severity, with cut-off values of 2.25 and 96.8 ml/L. RNA viral replication: no associations were found for SARS-CoV-2 replication and COVID-19 severity Patients with lower Shannon diversity displayed SARS-CoV-2 fecal replications 4 features: ↓ *Firmicutes*/*Bacteroidetes* ratio; ↑ *Proteobacteria* phylum; ↓ butyrate-producing bacteria from *Lachnospiraceae* family (*Roseburia* and *Lachnospira* genera) ↓ *Actinobacteria* essentially *Bifidobacteria* (*Collinsella*)
Wu et al. (54)	China	Longitudinal study for both (oral and fecal districts) during hospitalization from positive to negative virological cure Fecal samples and throat swabs V3–V4 of the 16S rRNA gene	-53 COVID-19 patients divided into 2 subgroups: non-severe COVID-19 (mild-moderate) and severe group (sever–critical) 73 controls Also, throat analyses Clinical features not shown	**α-Diversity** ↓ Faith in severe COVID-19 and non-severe COVID-19 subgroups compared to controls (with increased gradient among groups from severe to non-severe to controls) **β-Diversity** Separation among 3 groups (severe COVID-19, non-severe COVID-19 and controls) according to unweighted Unifrac	**LEfSe analysis** **Comparison between COVID-19 and controls** ↓ *Blautia*, *Coprococcus*, and *Collinsella* genera ↓ *Bacteroides caccae*, *Bacteroides coprophilous*, *Blautia obeum*, *Clostridium colinum* species ↑ *Streptococcus*, *Weisella*, *Enterococcus*, *Rothia*, *Lactobacillus*, *Actinomyces*, and *Granulicatella* genera ↑ *Clostridium citroniae*, *Bifidobacterium longum*, *Rothia mucilaginosa* species	*Granulicatella* and *Rothia* increased in both districts investigated (oral and gut) of COVID-19 patients. At the gut level, SARS-CoV-2 replication: +Correlation to *P. copri* and *E. dolichum* −Correlation to other taxa like *S. anginosus*, *Dialister*, *Alistipes*, *Ruminococcus*, *C. citronieae*, *Bifidobacterium*, *Haemophylus*, and *H. parainfluenzae* taxa SARS-CoV-2 infection associated with oral microbiome alterations In β-diversity: distinguishing ongoing antibiotics: both subgroups (with and without antibiotics) displayed different clusters compared to controls (but not between subgroups)
He et al. (55)	China	Longitudinal study until 3 months follow-up Fecal samples Multi-omics profiling (metaproteomics, glycoproteomics, metabolomics, lipidomics)	-13 COVID-19 patients with different severity index disease (7 mild, 5 moderates, 1 severe) 21 controls -Age 27 median but 2 patients < 3 years old (1 patient 1 year old, 1 patient 10 months), 1 patient 5 years old; controls 43 years old -Male 77%; controls 57% -BMI 24 with 2 obese patients and 1 underweight Comorbidities: 1 diabetic patient, 2 patients with sinusitis or rhinitis; several patients with gastrointestinal disorders and anorexia	**α-Diversity** No data **β-Diversity** Multiomics profiling confirmed the separation between COVID-19 and controls	**Relative abundance from the metaproteomic approach:** **COVID-19 vs. controls** ↓ *Lachnospiraceae* family (*Lachnoclostridium*, *Ruminococcus*, *Butyrivibio*, *Dorea*, *Blautia*, and *Tyzerella* genera) ↑ *Bacteroides* genus	Feature of this study: enrichment of gut bacteria-related deleterious metabolites as well as altered host and bacterial lipids.
Li et al. (56)	China	Cross-sectional study Fecal samples Shotgun metagenomic sequencing	-37 COVID-19 and 10 controls in the discovery cohort 10 COVID-19 and 9 controls in the validation cohort (controls matched for age, gender, and BMI. No antibiotics and/or probiotics 4 weeks before enrollment) According to the severity index: 7 mild, 29 moderate, 8 severe, and 3 critical (patients from both cohorts) -Age: 44-year-old patients and 37-year-old controls in discovery cohort; 56-year-old patients and 46-year-old controls in validation cohort -Men: 51% COVID-19 vs. 70% controls in the discovery cohort; 50% vs. 55% in the validation cohort BMI: 23 vs. 21 in the discovery cohort; 23 vs. 23 in the validation cohort ABT: 32% in the discovery cohort; 60% in the validation cohort Antiretroviral: 0% in the discovery cohort; 100% in the validation cohort Probiotic during hospitalization: 0% in the discovery cohort: 50% in the validation cohort	**α-Diversity** **Comparison between COVID-19 and controls** ↓ Number of species In the intragroup COVID-19 analysis according to the severity index: ↓ Evenness and Pielou indexes in mild type vs. controls **β-Diversity** Bray–Curtis separation	**Relative abundance comparison** **COVID-19 vs. controls** ↑ *Bacteroidetes* phylum and ↑ *Bifidobacterium longum*, *Streptococcus thermophilus*, and other taxa (note that several patients received probiotics, which include: *Bifibacterium longum Streptococcus thermophilus*, and *Lactobacillus bulgaricus*) ↑ *Bacteroides stercoris*, *Bacteroides vulgatus*, *Bacteroides massiliensis*, *Bifidobacterium longum*, *Streptococcus thermophilus*, *Lachospiraceae* bacterium, *Prevotella bivia*, *Erysipelotrichaceae* bacterium (2 variants) ↓ *Firmicutes* phylum ↓ *Candidatus saccharibacteria* taxa and *Corionacteriaceae* family ↓ *Ruminococcus*, *Dorea*, and *Adlercreutzia* genera ↓ *Clostridium nexile*, *Streptococcus salivarius*, *Coprococcus catus*, *Eubacterium hallii*, *Enterobacter aerogenes*, and *Adlercrutzia equolifaciens*	−Correlation between COVID-19 severity and *Rosebura* and *Megasphaer* genera −Correlation between COVID-19 severity and *Roseburia inulinivorans*, *Bacteroides faecis*, *Bifidobacterium bifidum*, *Parabacteroides goldsteinii*, *Lachnospiraceae* bacterium, and *Megasphaera* species +Correlation between *Paraprevotella*, *Lachnospiraceae*, *Erysipelotrichaceae* taxa, and COVID-19 severity +Correlation between *Paraprevotella* species, *Streptococcus thermophilus*, *Clostridium ramosum*, and *Bifidobacterium animalis*
Liu et al. (57)	China	A prospective, multicentered pilot study with a 6-month follow-up after hospital discharge (after virological clearance) Fecal samples shotgun metagenomic sequencing	-68 patients (from 106 enrolled) followed up from admission to 6 months 68 non-COVID controls Post-acute COVID-19 symptoms (PACS): at least 1 persistent symptom 4 weeks after clearance → *N* = 50/68 at 6 months Severity of COVID-19: most patients had mild to moderate severity of COVID-19 (81.1%) -Age, 48 years old -Men: 47% -BMI: no data Comorbidities (45%): hypertension is the most common comorbidity followed by type 2 diabetes mellitus ABT 23% but analyses on antibiotic-naïve patients Antiviral: 52% LPV/RTV, 28%RBV, 36% INF, 5% remdesivir Symptoms 6 months: fatigue, poor memory, hair loss, anxiety, difficulty sleeping They documented dietary records during the time of hospitalization Exclusion criteria for non-COVID-19 controls were the use of antibiotics in the past 6 months, the use of laxatives or antidiarrheal drugs in the past 3 months, and recent dietary changes	**α-Diversity** Longitudinal comparison from baseline to 6 months and vs. controls ↓ Shannon diversity and Chao-1 richness at 6 months compared to controls ↓ Shannon diversity and richness at admission in patients who developed PACS compared to controls **β-Diversity** Separation among groups: basal COVID-19 naïve antibiotic patients (and overall), longitudinal time points (1 month and 6 months with essential overlap), and controls No differences between COVID-19-naïve antibiotic patients and antibiotic patient subgroups during follow-up	**LEfSe analysis** **Longitudinal COVID-19 subgroups vs controls** ↓ *Ruminococcus* and *Bifidobacterium* (at 1 and 6 months compared with controls) and other taxa. When the effect of antibiotics was examined at baseline and at 6 months, overall gut microbiota composition was similar between antibiotic-naïve and antibiotic-treated patients. Whereas the overall gut microbiota composition was distinct at 1 month PACS analysis: patients who referred at least 1 COVID-19 symptom at 6 months (76%) maintained a different gut microbiota composition characterized by: ↑ *Ruminococcus gnavus*, *Bacteroides vulgatus*, *Bacteroides thetaiotaomicron*, *Lachnospiraceae* bacterium oral taxon, *Bacteroides xylanisolvens*, *Parabacteroides distasonis*, *Clostridium innocuum*, *Flavonifractor plautii*, *Lactobacillus delbrueckii*, Er*ysipelatoclostridium ramosum*, *Morganella morganii*, *Lactobacillus acidophilus*, *Streptococcus lutetiensis* ↓ *Faecalibacterium prausniztii*, *Collinsella aerofaciens*, *Eubacterium rectale*, *Blautia obeum*, *Ruminococcus torques*, *Ruminococcus bicirculans*, *Roseburia faecis*, *Adlecreutzia equolifaciens*, *Coprococcus comes*, *Dorea longicatena*, *Firmicutes* bacterium CAG-83, *Agathobaculum butyriciproducens*, *Dorea formicigenerans*, *Eubacterium* sp CAG-251, *Roseburia inulinivorans*, *Ruthenibacrerium lactatiformans*, *Gemigger formicilis*, *Enterococcus avium*, *Roseburia hominis*, *Ruminococcus lactaris*	The first study to demonstrate persistent gut dysbiosis at 6 months after recovery from COVID-19 and the link between altered gut microbiota and common lingering symptoms. Specific gut microbiome profiles were associated with the presence of PACS and with different PACS symptoms +Correlation between PACS patients with respiratory symptoms and opportunistic pathogens +Correlation between the abundance of nosocomial pathogens with neuropsychiatric symptoms and fatigue −Correlation between the relative abundance of multiple bacterial species beneficial to host immunity and the presence of PACS at 6 months −Associations of walking distance test with pathogenic bacteria species +Correlation between walking distance and several short-chain fatty acids and butyrate producers. No significant correlations between viral load and PACS development.
Ng et al. (58)	China	Prospective observational study Fecal samples Shotgun metagenomic sequencing	-138 adults who have received 2 doses of either the inactivated vaccines (CoronaVac; *n* = 37) or the mRNA vaccine (BNT162b2; *n* = 101) -Age; 47 years -Men: 32.6% -BMI 38.4% were classified as OWOB (i.e., BMI ≥ 23). It is a study to determine whether baseline gut microbiome composition was associated with the immune response to COVID-19 vaccines	**α-Diversity** ↓ At 1 month after the second dose of vaccination compared with baseline samples in both vaccine groups **β-Diversity** Shift at 1 month after the second dose of vaccination compared with baseline samples in both vaccine groups	At the species level: ↑ *Bacteroides cacca*e in CoronaVac vaccinees ↑ *Bacteroides caccae* and *Alistipes shahii* in BNT162b2 vaccinees ↓ Common bacterial species including *Adlercreutzia equolifaciens*, *Asaccharobacter celatus*, *Blautia obeum*, *Blautia wexlerae*, *Dorea formicigenerans*, *Dorea longicatena*, *Coprococcus comes*, *Streptococcus vestibularis*, *Collinsella aerofaciens*, and *Ruminococcus obeum* CAG 39 were observed in both vaccine groups ↓ *Actinobacteria* and *Firmicutes* Note: None of the participants reported significant dietary changes during the study period. Among 72 randomly selected participants, no significant changes in detailed dietary intake were recorded at baseline and 1 month after the second dose of vaccination Note: BNT162b2 → Comirnaty	CoronaVac vaccinees: -21/37 (56.8%) showed sVNT (surrogate virus neutralization test) lower than 60% (low responders). Distinct baseline gut microbiome from those with sVNT higher than 60% (high responders). *Bifidobacterium adolescentis* was enriched in high responders while *Bacteroides vulgatus*, *Bacteroides thetaiotaomicron*, and *Ruminococcus gnavus* were more abundant in the low responder. BNT162b2 vaccinees: Similar to CoronaVac, low responders had a persistently low level of *Actinobacteria*, particularly *B. adolescentis*. 4 specific bacteria in the baseline gut microbiome, including *Eubacterium rectale*, *Roseburia faecis*, and 2 *Bacteroides* species, *B. thetaiotaomicron*, and *Bacteroides* sp. OM05-12 were significantly increased in the highest-tier responders with the top 25% of sVNT level

ICU, Intensive Care Unit; BSI, bloodstream infections; OWOB, overweight and obese; sVNP, surrogate virus neutralization test; UPLC-MS, ultra-performance Liquid chromatography-mass spectrometry; HCWs, healthcare workers; HCs, healthy controls; SCFAs, short-chain fat acids; BPBs, butyrate-producing bacteria; upward arrows “↑”, increase; downward arrows “↓”, decrease. In correlation and other findings, “−” and “+” means respectively negative and positive correlation.

To critically revise the studies, we first considered all the variables potentially influencing the final observations: study design, location, material source, microbial technology used, sample size, and patient characteristics—age, body mass index (BMI), gender, sexual behaviors, COVID-19 severity index, comorbidities, recent previous use of antibiotics/probiotics, diet, and lifestyle.

The cross-sectional study design was the most common. Less than half of studies (45%) had a longitudinal/prospective design, 20% of which focused on long-COVID-19.

The study location was a critical factor: most studies (19/22, 86%) were set in Asia (18 in China, one in South Korea), and three of 22 (14%) in Europe; no other geographic regions were represented.

Lifestyle and diet were not analyzed, even though both factors are crucial elements in shaping microbial core composition (32, 59, 60).

The material source was a fecal sample in 19/22 (86%) studies, while three of 22 (14%) were based on anal swab analysis. Most studies (12/22) used next-generation sequencing (NGS) technology through ribosomal-S16-DNA hypervariable region sequencing (V4 or V3–V4 regions preferred) to analyze microbiota; shotgun metagenomic sequencing was used in seven of 22 studies, whereas one study used multi-omics methodologies (55), one study nanopore technology (38), and another used quantitative PCR (39).

Regarding patients’ characteristics, all studies included both men and women, but no studies considered sexual behavior, although its impact on microbiota core is known in several disease models (61, 62). Only one-third of studies (seven of 22) included BMI data, and control groups, when included, were often matched for BMI. Fifty percent of the subjects in the studies, 50% were aged 50 or younger.

The small sample size was a limit reported by several authors, with a total number of enrolled subjects below 40 in almost two-thirds of studies 13/21 (62%). The COVID-19 severity index was reported by most studies, with high heterogeneity in the works analyzed.

Scarce data were available on comorbidities and concomitant medications; hypertension was the most commonly reported, followed by diabetes.

No data were generally reported on COVID-19 vaccine status for subjects enrolled after the introduction of the vaccine; only one study investigated the microbiota changes in two groups of patients vaccinated with two different vaccines (58). During hospitalization, both antibiotics and/or antiretroviral treatments and probiotics were administered in several studies; however, these data were not critically investigated in most published studies.

### 2.2 Microbiota analysis

After assessing the possible confounding factors, we compared the gut microbiota features according to two ecological measures, α-diversity and β-diversity, in association with relative abundance results.

In humans, α-diversity measures the level of diversity within individual samples; it includes several indexes gathered in two groups: richness indexes (Faith index, Observed and Chao-1 index) and evenness indexes (Shannon index, Peliou’s evenness, Simpson, and inverse Simpson indexes) (63, 64).

In parallel to other disease models, α-diversity at the gut level, more frequently described with richness indexes (like Chao-1), resulted in a global reduction in all COVID-19 patients compared to controls (see details in **Table 1A**). An interesting study observed this reduction already in the acute phase of the disease (48). On the contrary, Yeoh et al. (43) did not report alterations in α-diversity indexes, even though they enrolled most COVID-19 patients with a mild or moderate severity index (90% of patients).

In a Korean longitudinal analysis performed on patients who were asymptomatic or affected by the mild disease, an increase in α-diversity (Peliou’s evenness) was observed in the recovered subgroups compared to infected patients (51). Interestingly, Xu et al. (46) observed a trend toward increased bacterial diversity from the early to late stages of COVID-19 in a 35-day longitudinal analysis of inpatients with mild disease. Furthermore, the same study described an interesting synchronous restoration of microbiota in both gut and upper airways, suggesting a possible role of the gut-lung axis.

Moreira-Rosario et al. (53) described a reduced α-diversity gradient trend (Shannon index) from mild to severe COVID-19 patients, and Chen et al. (48) showed how richness was not restored to a normal level even after 6 months in 30 COVID-19 patients (one-third with severe disease), although a trend toward healthy controls was noticed.

β-Diversity measures the level of diversity (or dissimilarity) between samples, mostly by using a Permanova analysis (65, 66). All the studies showed a difference between COVID-19 patients and controls, in general, and according to different severity index categories.

Mazzarelli et al. (44) have shown a difference in β-diversity among patients hospitalized in regular wards compared to ICU patients and hospitalized no-COVID-19 controls, although no data on prior antibiotic intake was gathered. Regarding this aspect, two studies (9, 43) compared microbiota composition in COVID-19 patient subgroups (with and without antibiotics) with healthy controls, confirming a separation among groups, with high heterogeneity revealed in the antibiotic subgroup.

Regarding relative abundance analysis, several studies described a significant reduction in *Firmicutes* members, especially for BPBs (both *Lachnospiraceae* and *Ruminococcaeae* families, mostly *Faecalibacterium prausnitzii*) in COVID-19 patients compared to no-COVID controls, while discordant data have been reported about *Erysipelotrichaceae* and *Veillonellaceae* taxa.

Conversely, several facultative anaerobic bacteria like members of the *Bacilli* class, resulted in increased growth, mostly in the *Enterococcaceae* family as well as *Streptococcaceae* and *Lactobacillaceae* (**Table 1**). Contrasting data have been described regarding the *Bacteroidetes* phylum during COVID-19, with some works reporting an increase in *Bacteroidetes* phylum with a consequent reduction of the *Firmicutes*/*Bacteroidetes* ratio (53) as opposed to other studies reporting a reduction in taxa belonging to this phylum. Other factors, like diet and/or antibiotics, could play a role in these findings, highlighting the importance of assess for confounding factors when considering the study results.

Reduction in the *Actinobacteria* phylum, including the *Bifidobacterium* genus and *Collinsella* genus (recently associated with SARS-CoV-2-ACE2 binding inhibition), represents another significant finding in COVID-19 studies (67). The *Bifidobacterium* genus was found to be increased only in three studies (notably, in one study, a probiotic including this taxon was administered (56)), while the *Collinsella* genus resulted was increased in a few other studies (40, 45, 49); the reason for this last difference is not clear. *Proteobacteria* resulted increased in almost all studies performed on COVID-19 patients, although some authors have described an increase in *Enterococcaceae*/*Enterobacteriaceae* ratio (39), probably linked to the use of antibiotics. Finally, the *Akkermansia* genus (*Verrucomicrobia*), a propionate-producing bacterium genus with anti-inflammatory features, resulted in reduced COVID-19 (but not in all studies). To note, the severity of COVID-19 disease seems to emphasize differences in the relative abundance of gut microbiota, although most studies included asymptomatic/mild/moderate categories.

## 3 Airway microbiota dysbiosis in acute COVID-19

We analyzed 13 studies on airway microbiota changes during SARS-CoV-2 infection, mostly comparing COVID-19 patients with healthy subjects and/or patients with different respiratory diseases (**Table 1B**).

**Table 1B:** Selected studies on airway microbiota and COVID-19.

ID	Country	Study characteristics	Population characteristics	α/β-diversity	Microbiome modifications: relative abundance analyses	Correlations and other findings
De Maio et al. (68)	Italy	Cross-sectional study Nasopharyngeal swab Amplification V1–V2–V3 regions of the bacterial 16S rRNA	40 patients; 18 with confirmed SARS-CoV-2 infection, 22 HCs	No difference (observed species, Shannon index, and inverse Simpson)	Most sequences in all samples (98% in both SARS-CoV-2 and HCs) belonged to 5 phyla: *Firmicutes* (42% and 51%, respectively), *Bacteroidetes* (25% and 20%, respectively), *Proteobacteria* (18% and 16%, respectively), Actinobacteria (8% and 6%, respectively), and *Fusobacteria* (5% and 5%, respectively)	
Rueca et al. (69)	Italy	Cross-sectional study Nasal and oropharyngeal swabs Amplification V1–V2–V3 regions of the bacterial 16S rRNA	39 patients, 21 with confirmed SARS-CoV-2 infection; 8 affected by a different human coronavirus (HKU, NL63, and OC43); 10 HCs Disease severity: critically ill (ICU) vs. paucisymptomatic (Pauci)	Chao-1 decreased SARS-CoV-2 ICU as compared to SARS-CoV-2 Pauci patients, other HCoVs and HCs Shannon index decreased in SARS-CoV-2 ICU patients compared to HCs and SARS-CoV-2 Pauci patients	At the phylum level: -*Deinococcus Thermus* was present only in controls as compared to SARS-CoV-2 ICU patients, SARS-CoV-2 Pauci, or other HCoV patients - *Candidatus Saccharibacteria* (TM7) was strongly increased in negative controls and SARS-CoV-2 Pauci patients as compared to SARS-CoV-2 ICU patients and Other HCoV patients At the family level: - *Alicyclobacillaceae*, *Chromobacteriaceae*, *Deinococcacaee*, *Hydrogenophilaceae*, *Thermoanaerobacteraceae*, *Sporomusaceae*, and *Thermoanaerobacterales* family III. Incertae Sedis were exclusive microorganisms detected in neg control patients-*Pectobacteriaceae* were exclusive to SARS-CoV-2 ICU patients At the lower taxonomic level: -*Johnsonella*, *Tepidiphilus*, *Thermoanaerobacter*, *Thermoanaerobacterium*, *Thermosinus*, and *Variovorax* were exclusive to neg control patients -*Salmonella*, *Scardovia*, *Serratia*, and unk_*Pseudomonadaceae* were included exclusively in SARS-CoV-2 ICU patients	SARS-CoV-2 ICU patients displayed a complete depletion of *Bifidobacterium* and *Clostridium* The presence of *Moraxellacaea* spp. was observed exclusively in SARS-CoV-2 Pauci patients The presence of *Pseudomonaceae* was found exclusively in SARS-CoV-2 ICU
Shen et al. (70)	China	Cross-sectional study BALF RNA extraction, reverse-transcripted, amplified	53 patients, 8 with confirmed SARS-CoV-2 infection; 25 with CAP, and 20 healthy controls	Significative lower in patients with pneumonia (both COVID-19 and CAP)	3 types of microbiotas: -Type I dominated by the possible pathogens -Type II were mostly environmental organisms (contamination) -Type III mainly commensal species	
Nardelli et al. (71)	Italy	Cross-sectional study Nasopharyngeal swab Amplification V1–V2–V3 regions of the bacterial 16S rRNA	38 patients, 18 with confirmed SARS-CoV-2 infection; 20 HCs	No difference (Chao-1: *p* = 0.28, Shannon: *p* = 0.27, and Simpson: *p* = 0.32)	5 phyla prevalent in both HCs and COVID-19: - *Firmicutes*, *Bacteroidetes*, *Actinobacteria*, *Proteobacteria*, and *Fusobacteria* In COVID-19: -Significant lower abundance of *Proteobacteria* and *Fusobacteria* -At the genus level, reduced *Leptotrichia*, *Fusobacterium*, and *Haemophilus*	Negative correlation between the relative abundance of *Fusobacterium periodonticum* and the severity of the patient’s symptoms
Budding et al. (72)	The Netherlands	Cross-sectional study Throat swab Differentiation of species by length polymorphisms of the 16S-23S rDNA region combined with phylum-specific sequence polymorphisms of the 16S rDNA	135 patients, 46 with confirmed SARS-CoV-2 infection, 89 HCs	No data	A cluster of 77 samples with a similar microbiota composition (both HCs and COVID-19) with a high abundance of *Haemophilus parainfluenzae*, *Neisseria cinerea*, *Streptococcus mitis* group, *Streptococcus bovis* group, *Leptotrichia buccalis*, and *Rothia mucilaginosa*	
Ventero et al. (73)	Spain	Cross-sectional study Nasopharyngeal swab Amplification V1–V2–V3 regions of the bacterial 16S rRNA	74 patients, 56 with confirmed SARS-CoV-2 infection; 18 HCs	No data	Most abundant phylum: -*Firmicutes* (52.9% ± 4.0%) -*Bacteroidota* (22.1% ± 6.1%) -*Proteobacteria* (12.7% ± 7.3%) -*Actinobacteria* (5.4% ± 0.6%) At the genus level: -*Streptococcus* (25.2% ± 2.0%) -*Prevotella* (16.2% ± 5.7%) -*Veillonella* (14.4% ± 2.2%) -*Haemophilus* (5.23% ± 4.78%) -*Moraxella* (3.2% ± 3.6%) OTUs: -*Bacteroidota* (18) -*Firmicutes* (25)	The most common genera among the OTUs found exclusively on COVID-19-positive patients were *Prevotella* (13), followed by *Leptotrichia* (4) and *Streptococcus* Among the OTUs positively associated with COVID-19 severity, 3 were classified as members of the genus *Prevotella*, and 1 to a closely related genus, *Alloprevotella*
Rosas-Salazar et al. (74)	USA	Cross-sectional study Nasal swab Amplification V1–V2–V3 regions of the bacterial 16S rRNA	59 patients, 38 with confirmed SARS-CoV-2 infection; 21 HCs	Higher α-diversity in SARS-CoV-2 No differences in any of the measured β-diversity metrics between groups	HCs: -*Staphylococcus* (41.56%), *Corynebacterium*_1 (28.09%), *Moraxella* (8.48%), *Dolosigranulum* (3.56%), and *Neisseria* unclassified (1.98%) COVID-19: -*Corynebacterium*_1 (33.66%), *Staphylococcus* (29.34%), *Dolosigranulum* (5.29%), *Peptoniphilus* (3.91%), and *Lawsonella* (3.22%) COVID-19 with high viral load: -*Corynebacterium*_1 (35.69%), *Staphylococcus* (28.83%), *Peptoniphilus* (6.67%%), *Anaerococcus* (4.79%%), and *Bacteroides* (3.83%) COVID-19 with low viral load -*Corynebacterium*_1 (41.44%), *Staphylococcus* (20.75%), *Dolosigranulum* (12.30%), *Lawsonella* (4.50%), and *Peptoniphilus* (2.76%).	No correlation between SARS-CoV-2 viral load and diversity measures
Miao et al. (75)	China	Cross-sectional study BALF, ETA RNA and DNA extraction, reverse transcription, and use of DNA libraries	50 airway samples from 323 patients with confirmed SARS-CoV-2 infection	α-Diversity of critically severe COVID-19 patients is lower than non-intubated patients but similar to intubated non-COVID-19 group PCoA analysis: the greatest difference between non-intubated patients versus the other 2 groups with intubation	Higher relative abundance in COVID-19: -*Acinetobacter*, *Klebsiella*, *Pelomonas*, *Ralstonia*, and *Sphingomonas* Lower relative abundance in COVID-19: -*Actinomyces*, *Haemophilus*, *Neisseria*, *Prevotella*, *Streptococcus*, and *Veillonella*	
Braun et al. (76)	Israel	Cross-sectional study Nasopharyngeal swab Amplification V1–V2–V3 regions of the bacterial 16S rRNA	33 patients with confirmed or suspected SARS-CoV-2 infection	No difference in α-diversity (faith’s phylogenetic diversity, Shannon) and evenness (Wilcoxon rank sum test) Unweighted Unifrac-based PCoA: no clustering by COVID-19 test results	No cluster identified	
Zhang et al. (77)	China	Cross-sectional study Nasopharyngeal swab and sputum RNA isolation, reverse transcription with N6 random primers after adaptor ligation with T4 ligase and library amplification, sequencing	187 patients, 62 with confirmed SARS-CoV-2 infection; 125 HCs	Shannon diversity index in sputum samples is significantly lower in COVID-19 cases	31 species in nasopharyngeal samples and 178 species in sputum samples with different abundance between COVID-19 and non-COVID-19 cases Most species less abundant in COVID-19 cases	
Mostafa et al. (78)	China	Cross-sectional study Nasopharyngeal swab cDNA sequencing for sequencing poly(A) RNA full-length transcripts	50 patients; 40 with confirmed SARS-CoV-2 infection; 10 with suspected SARS-CoV-2 infection Each patient was assigned a 4-point severity index according to the clinical presentation	Lower diversity in COVID-19 (Shannon diversity index, Chao-1 richness estimate, Simpson diversity)	*Propionibacteriaceae* are proportionately more abundant in COVID-19 *Corynebacterium accolens* decreased in COVID-19	
Merenstein et al. (79)	USA	Longitudinal study Oropharyngeal, nasopharyngeal, ETA, BALF Amplification V1–V2–V3 regions of the bacterial 16S rRNA	83 patients with confirmed SARS-CoV-2 infection; 42 HCs	Lower diversity in COVID-19	Upper airway microbiota comparison between COVID and HCs: -COVID-19 patients lower abundance of *Proteobacteria*, a greater abundance of *Bacteroidetes* Association with disease severity: -Different microbiota between COVID-19 patients with moderate/severe (WHO 4–6) and critical/fatal outcomes (WHO 7–10) -Decreased oropharyngeal *Proteobacteria* and *Actinobacteria* correlated with greater WHO score over the course of hospitalization -At the genus level, patients with more severe disease had significantly lower relative abundances of *Hemophilus*, *Actinomyces*, and *Neisseria*, all of which are abundant in the normal oropharyngeal microbiome	α-Diversity in oropharyngeal samples at the first time point correlated with COVID-19 severity, with lower diversity associated with higher severity The rate of change in oropharyngeal bacterial community structure was significantly greater in COVID-19 than in non-COVID subjects
Xu et al. (46)	China	Longitudinal study Throat swab Amplification V1–V2–V3 regions of the bacterial 16S rRNA	64 patients, 35 with confirmed SARS-CoV-2 infection, 10 with other diseases	Decrease in α-diversity, significantly lower richness and evenness in COVID-19	HCs: -Prevalence of genus *Bacteroides* and unclassified *Comamonadaceae* COVID-19, 4 community types, with a progressive imbalance of microbiota: -Type 1: *Alloprevotella* -Type 2: *Porphyromonas*, *Neisseria*, *Fusobacterium*, and unclassified *Bacteroidales* -Type 3: *Pseudomonas* -Type 4: *Saccharibacteria incertae sedis*, *Rothia*, and unclassified *Actinomycetales*	Among 22 COVID-19 adults who had specimens at 2 or more timepoints, over half (12, 54.5%) maintained a relatively stable microbiome community types

ICU, Intensive Care Unit; HCs, healthy controls; BALF, bronchoalveolar lavage fluid; CAP, community acquired pneumonia; OTU, operational taxonomic unit; ETA, endotracheal aspirate.

Nasopharyngeal swabs were the most studied material, with the exception of three studies analyzing samples from the lower respiratory tract, such as bronchoalveolar lavage fluid and endotracheal aspirate. Bacterial communities were prevalently mapped by amplification of 16S gene hypervariable regions, with only a few studies employing genome sequencing. Eighty percent of the studies were set in China or Europe (five studies each). No data on possible confounding factors such as diet, BMI, relevant comorbidity, and antibiotic/antiviral consumption were investigated.

Overall, patients with SARS-CoV-2 infection showed diminished diversity in airway microbiota composition, by means of Shannon, Simpson, and Chao-1 indexes, when compared to both healthy subjects (46, 69, 70, 75, 77–79) and patients with community-acquired pneumonia (70).

A similar reduction in diversity measures is reported in critically ill COVID-19 patients, as opposed to subject with milder symptoms, other coronavirus infections, and healthy subjects (69). Interestingly, a reduction in diversity and greater difference at principal coordinate analysis (PCoA) is observed in patients needing mechanical ventilation compared to non-intubated patients regardless of SARS-CoV-2 infection (75). Such data suggest that COVID-19 impacts airway microbiota diversity mostly in severe infections, and this imbalance is strongly biased by other confounding factors such as intubation.

Of note, a number of the report showed no significant differences between COVID-19 patients and the control group in both bacterial richness and diversity/evenness indexes (observed species, Shannon index, and inverse Simpson index) (68, 71, 76). These findings can be partially explained by the heterogeneous population included in the studies and by the different methods used to sequence bacterial communities and assess diversity.

Curiously, Rosas-Salazar et al. (74) observed higher overall α-diversity in SARS-CoV-2-infected subjects compared to healthy controls, with no significant differences in any of the measured β-diversity.

COVID-19 severity correlates to α-diversity in oropharyngeal samples at the first time point, with lower diversity associated with higher disease severity (79). However, no significant association between high versus low SARS-CoV-2 viral load and any of the α-diversity or β-diversity metrics was observed (74).

In the studies analyzed, the airway microbiota of healthy individuals is characterized by the predominance of *Bacteroidetes* and *Comamonadaceae* taxa (46, 68), and no specific microbiota pattern has been found in COVID-19 patients. However, some peculiar alterations in relative composition have been observed.

Reduced abundance in *Proteobacteria* and *Fusobacteria* phyla is reported in subjects with SARS-CoV-2 infection as compared to controls, and decreased oropharyngeal *Proteobacteria* and *Actinobacteria* phyla correlate with greater disease severity (71, 79). At the genus level, patients with more severe diseases have significantly lower relative abundances of *Haemophilus*, *Actinomyces*, and *Neisseria*, all of which are abundant in the normal oropharyngeal microbiome (74, 79). Interestingly, *Fusobacterium periodonticum* is less represented in COVID-19 patients, negatively correlating with the severity of symptoms (71). A possible explanation is that these bacteria could modulate sialic acid metabolism and regulate ACE expression, impacting SARS-CoV-2 binding to the epithelium of the respiratory tract, as shown for other intestinal microorganisms (71, 80).

Conversely, COVID-19 patients show a high abundance of *Saccharibacteria* (formerly known as TM7), *Streptococcus mitis* group, *Streptococcus bovis* group, and *Rothia mucilaginosa* taxa (46, 72, 73), the latter often associated with cancer and bacteremia (81).

Significant changes among operational taxonomic unit (OTU) abundances are also reported, with decreased complexity of coabundance networks in severe COVID-19. OTUs associated with higher disease severity are members of the genus *Prevotella* and *Veillonella*. Particularly, it has been postulated that *Prevotella* spp. can worsen disease progression by activating immune signaling pathways that modulate inflammation (73).

Critically ill COVID-19 patients display a complete depletion of *Bifidobacterium* and *Clostridium* genera, with the presence of *Salmonella*, *Scardovia*, *Serratia*, and *Pectobacteriaceae* taxa. In these subjects, there is also a relative abundance of the *Pseudomonaceae* family, known to be associated with pathogenic conditions such as severe acute respiratory syndromes (69). Another characteristic of the airway microbiota in severe COVID-19 patients is low diversity and more richness in non-fermenting bacteria like *Acinetobacter*, *Pelomonas*, *Ralstonia*, and *Sphingomonas* genera. As mentioned before, these changes might be attributed to intubation and mechanical ventilation rather than COVID-19 pneumonia per se (75).

Interestingly, similar characteristics of an imbalanced microbiota with an enrichment of proinflammatory *Enterobacteriaceae* are found in patients with other respiratory diseases (46).

To date, there is scarce data coming from longitudinal studies on airway microbiota in SARS-CoV-2 infection. Analyzing throat swabs from 64 patients, 35 of which with confirmed infection, Xu et al. (46) postulated that a peculiar microbial community might represent the progressive imbalance of the respiratory microbiota. Interestingly, even though over half COVID-19 patients analyzed maintained relatively stable microbiome community types, 70% of the subjects experienced a gradual decrease of microbial diversity, with the enrichment of opportunistic pathogenic bacteria such as *Saccharibacteria* and *Rothia* and a reduction of *Alloprevotella*. This shift toward dysbiosis shows how impaired homeostasis of inflammation pathways, a hallmark of the advanced stage of SARS-CoV-2 infection, affects microbial communities and can represent a biomarker of disease progression.

## 4 Microbiota dysbiosis in long-COVID

### 4.1 Microbiota changes in long-COVID

Few studies tried to investigate α-diversity alterations during long-COVID: in this setting, Zhuo et al. (52) reported a reduced Shannon index in a 15-patient cohort, followed up for 3 months with at least one persistent COVID-19 symptom. Coherently with these findings, in a 6-month follow-up, Liu et al. (57) have confirmed in long-COVID patients both a persistently reduced α-diversity (Shannon and Chao-1 indexes) and different gut microbiota clusters compared to controls. Notably, the subgroup who had COVID-19 at baseline without developing long-COVID did not show the same dysbiosis pattern. Reduced BPBs were reported in both COVID-19 subgroups compared to controls, but only in the long-COVID subgroup the microbial composition was different compared to controls at 6-month follow-up (**Table 1A**). Interestingly, the authors found no correlation between viral load in the gut and respiratory levels and long-COVID development at 6 months, nor did they find any effect of previous antibiotic intake. On the contrary, in the long-COVID subgroup, increased fecal relative abundance of opportunistic pathogens was positively associated with fatigue, respiratory and neuropsychiatric symptoms, while decreased other anti-inflammatory/BPB taxa was negatively correlated with long-COVID at 6 months. Coherently, Zhuo et al. (52) described both a negative correlation between some taxa (*Faecalibacterium prausnitzii*, *Intestinimonas butyriproducens*) and chronic respiratory symptoms as well as a positive correlation between *Proteobacteria* members and long-COVID symptoms.

### 4.2 Microbiota role in neurological and pulmonary symptoms

Persistent dysbiosis in long-COVID and its pathogenic role still need to be studied in humans, while rodent and non-human primate animal models of COVID-19 already showed long-term changes in both lung and gut microbiome (82, 83). The influence of gut microbiota on neurological symptoms, *via* the gut-brain axis, has been investigated in the animal model since the early decades of the new millennium. In murine models, Bercik at al. suggested that gut microbiota could influence the behavior of mice (84). Recently, Carloni et al. identified a closing in the choroid plexus vascular barrier during gut inflammation, suggesting a link between intestinal inflammation and neurologic/psychiatric symptoms, like a deficit in short-term memory and anxiety-like behavior (85). Moreover, a recent review summarized three different arms of inflammation for the gut-brain axis in a non-COVID-19 setting, where the systemic humoral pathway, cellular immune pathway, and neuronal pathway are involved (86). By translating these inflammatory patterns to the long-COVID setting, where gut dysbiosis persists at least after 6 months of follow-up, we can conclude that this microbial imbalance plays a role in maintaining both a chronic inflammatory status at the gut level and favoring the development of neurological/neuropsychiatric symptoms, as seen in the animal models mentioned above. However, it is not clear which immunologic pathway is dominant during long-COVID. It is plausible that several factors could coexist in the same disease model: (a) reduction in BPBs leading the butyrate loss linked to neuropsychiatric disorders (87); (b) development of the cytokine release syndrome during COVID-19, in particular with increased kynurenine:tryptophan ratio, already linked to depression syndrome (88); and (c) changes in l-DOPA production, regulated by ACE2 activation at the gut level (89).

There is still a lack of evidence on the role of microbiota dysbiosis in respiratory symptoms during long-COVID. Shortness of breath, frequently experienced by subjects after recovery from primary SARS-CoV-2 infection, could represent a clinical manifestation of the fibrosis secondary to chronic inflammation of lung parenchyma, leading to reduced total lung capacity. Such a condition is already linked to gut dysbiosis in non-COVID patients, as described in a recent review (90).

## 5 Relationship between gut dysbiosis, fecal SARS-CoV-2 replication, and immune-inflammation in COVID-19

It is well known that some microbial species can modulate ACE2 receptor expression and/or prevent SARS-CoV-2-ACE2 binding (67). Moreover, some studies found that the gut microbiota composition of COVID-19 patients, especially during hospitalization, is correlated with plasma concentrations of several cytokines, chemokines, and inflammation markers, suggesting that the gut microbiota could play a role in modulating host immune response and potentially influence disease severity and outcomes (43).

Interestingly, Zhuo et al. (50) studied α-diversity in a COVID-19 cohort stratified according to the presence of fever, discovering that COVID-19 patients with fever have shown a trend in reduced Chao-1 index compared to patients without fever, and similarly a β-diversity separation measured with Bray–Curtis. A negative correlation between PBPs and both inflammatory markers (9, 39, 43) and viral gut SARS-CoV-2 replication (40) was reported, despite the presence of GI disease and/or virological clearance. Interestingly, Zuo et al. (9) have discovered a negative correlation between *Bacteroides* taxa and fecal SARS-CoV-2 load and a positive correlation between *Erysipelotrichaceae* taxa and fecal SARS-CoV-2 replication. In contrast, Moreira-Rosario et al. (53) failed to see an association between fecal RNA viral replication and COVID-19 severity.

Wu et al. (46) reported a positive correlation between fecal SARS-CoV-2 replication and *P. copri*, *E. dolichum* taxa and a negative correlation between SARS-CoV-2 replication and other taxa like *Streptococcus*, *Dialister*, *Alistipes*, *Ruminococcus*, *Clostridium*, *Bifidobacterium*, and *Haemophylus* genera.

Finally, a longitudinal interventional study implementing fecal microbiota transplantation (FMT) in COVID-19 (45) described modulation of both gut microbiota core and peripheral lymphocyte subsets, with an increase in healthy taxa associated with a reduction in peripheral naïve B cells and an increase in memory B cells.

Data coming from clinical trials enrolling COVID-19 patients analyzing other possible drugs modulating gut microbiota, such as probiotics, are still scarce and not conclusive (91).

## 6 Conclusion

Microbiota homeostasis plays a role in human health and disease, and that applies to SARS-CoV-2 infection as well. During the last 2 years, several studies reported dysbiosis in COVID-19 patients for both gut and lung microbial composition. The main microbiota alterations that have been observed during COVID-19 were (a) significant reduction in α-diversity, already during the early phase of the disease and especially at the gut level, with a gradient from mild to severe clinical categories; (b) different β-diversity composition of microbiota core, characterized by a profile with higher facultative anaerobic bacteria and lower obligate anaerobic bacteria; and (c) possible connections between gut dysbiosis and peripheral inflammation markers, such as cytokines.

Data from longitudinal analyses currently available do not clearly show whether gut dysbiosis in COVID-19 ends with a complete functional restoration or if it does persist, posing the physiopathological premises for long-COVID. Indeed, a prolonged alteration of gut microbiota following the primary infection could contribute to causing some of the neurological and respiratory symptoms reported *via* the gut-brain and gut-lung axis. Further longitudinal studies are needed to characterize these conditions and assess the impact of prior comorbidity on the natural history of dysbiosis in SARS-CoV-2 infection.

Moreover, a knowledge gap regarding the role of FMT and other therapeutic approaches emerged, reinforcing the necessity for new evidence on the interaction of microbiota with host immunity. Such information is paramount to developing microbiota interventions aimed at improving COVID-19 and long-COVID outcomes.

## Author contributions

Conceptualization: GA, LA, EP, and AB. Data analysis: GA, LA, EP, AT, and AP. Editing and supervision: AM, CA, AG, and AB. All authors have read and agreed to the published version of the manuscript.
